# A Clinical Audit of the Assessment and Management for Those Diagnosed With Young Onset Dementia Within the Shepway CMHSOP

**DOI:** 10.1192/bjo.2024.420

**Published:** 2024-08-01

**Authors:** Rachel Rice

**Affiliations:** KMPT, Kent, United Kingdom

## Abstract

**Aims:**

To audit the Shepway CMHSOP against the NICE guidelines in dementia and the Royal College of Psychiatrists recommendations for service provision in young onset dementia.

**Methods:**

Data was collected retrospectively for all patients open to CMHSOP within the last 2 years with a diagnosis listed as dementia under the age of 65 years old.

**Results:**

The work up prior to diagnosis met some standards but improvements could be made in other areas. Mood was considered in all patients. The majority of patients (89%) had young onset blood tests if there was a clinical indication. However physical examination was only carried out in 43% of patients. In addition to this where physical examination was completed it was often limited to a brief note about the patient's gait and tremor.

Imaging standards were met within the Shepway CMHSOP with all patients having a scan, some patients being referred for additional specialist scans where indicated. There is also a neuroimaging MDT in which scans can be discussed with a neuro-radiologist.

The follow up care and support was an area that needs further development within Shepway CMHSOP. There is no named lead for those diagnosed with young onset dementia. Furthermore, only half of patients received a named practitioner to support their care. In addition to this only 79% were offered cognitive stimulation therapy and post diagnostic support which incorporate education for the carers. It is difficult to know if these options were discussed and declined by the patients, but if this is the case it would have been good practice to document.

**Conclusion:**

The time from referral to diagnosis was similar in those with a dementia with a well established and clear subtype (Down syndrome) to those diagnosed with other types of young onset dementia, 6 months and 5.5 months respectively.

My audit identified areas for improvement in the workup to diagnosis and the aftercare to support those diagnosed and their carers in order to meet NICE guidelines and the Royal College of Psychiatrists recommendations for service provision in young onset dementia.

Shepway CMHSOP will develop a young onset dementia pathway to ensure those diagnosed are offered the appropriate investigations and support following their diagnosis in line with these guidelines.

